# Acupuncture for tinnitus immediate relief

**DOI:** 10.1016/S1808-8694(15)30053-7

**Published:** 2015-10-19

**Authors:** Daniel Mochida Okada, Ektor Tsuneo Onishi, Fernando Ioriatti Chami, Andrei Borin, Nicolle Cassola, Viviane Maria Guerreiro

**Affiliations:** a MD, acupuncturist, postgraduate student of Otology UNIFESP-EPM.; b MD, MS, PhD in Otorhinolaryngology - UNIFESP-EPM, Preceptor of Otorhinolaryngology UNIFESP-EPM.; c MD, MS, PhD in Otorhinolaryngology - UNIFESP-EPM, acupuncturist.; d MD, MS in Otorhinolaryngology UNIFESP-EPM, acupuncturist.; e UNIFESP-EPM Graduate, Director of the Student League of Otorhinolaryngology UNIFESP-EPM.; f UNIFESP-EPM Graduate, Director of the Student League of Otorhinolaryngology UNIFESP-EPM

**Keywords:** Tinnitus, Acupuncture, Treatment

## Abstract

The treatment of tinnitus, wich is defined as conscientious perception of a sound originated in the ears or nervous system, represents until the current days a great challenge. The use of Acupuncture (ACP) is based on the stimulation with needles of specific points on the human anatomy. A prospective, randomizaded and double-blinded study was carried through in 76 patients taken care of in the Clinic of Tinnitus of the Department of Otorhinolaringology and Head and Neck Surgery of the UNIFESP-EPM in the period understood between April and June of 2005. All the patients had humming complaint and had been submitted to clinical anamnese, physical examination and subsidiary exams in order to investigate its etiology. The patients then were directed to a first researcher that determined an initial numeric value of the humming through Visual Analoge Scale(VAS), varying from 0 to 10 points. After this, had been directed for another room in which an acupuncturist doctor, who did not have access to the initial evaluation, separated the patients in Group Control and Group Study according to the attendance order, in alternating way. The ACP point used in patients of the Group Study places 6,5 cm above of the apex of the auditory pavilion in the parietal region. The point used in the Group Control places 3 cm above of the previous point, in the same vertical line. Then they had been sent back to the initial room for a new evaluation by the first researcher, where they had been guided to redefine the subjective score of the humming. Among the 76 studied patients, 29 were male (38,2%) and 47 female (61,8%), with average age 56,9 + 12,0 years. The Groups Study and Control had counted on 38 patients each. Through the Anova test it was evidenced that it had significant difference (p<0,001) between the moments pre and post needling and that in the group Study this improvement is more evident (p=0,0127). The t-independent test showed that it had a significant difference (p=0,017) between the two moments in the groups Study and Control. We conclude that there was significant reduction of the counting of the moments pre and post needling in both the groups, and in the group study the reduction is greater that in the group control.

## INTRODUCTION

Tinnitus is defined as the conscious perception of a noise which originates in the ear or the nervous system. The treatment of this condition is still a challenge today. In certain situations the absence of a specific source complicates the specific diagnosis and treatment. The pathophysiology is complex and not well understood. A variety of etiologies has been suggested including otological, metabolic, cardiovascular, dental, neurological, spinal and psychiatric diseases and others related to taking drugs, caffeine, alcohol and smoking.

In 1996 the National Institute of Health stated that 15% of North-Americans suffered tinnitus and that this led to significant undesirable effects on their lives. A survey done at the Tinnitus Sector of the Otorhinolaryngology Out-Patient Unit at FMUSP demonstrated that 50% of these patients had sleep disorders, 43.5% had loss of concentration, 59% had emotional unbalances and 14% complained about problems in their social activities^1^.

We know that the treatment of tinnitus should be tailored individually but the subjectivity of symptoms and wide range of possible etiologies, frequently in the same patient, compromise results. There are many forms of treatment to eliminate or at least in most cases relieve symptoms, including drug therapy, Tinnitus Retraining Therapy, hearing aids, electrical stimulus with cochlear implants, biofeedback and psychotherapy.

Acupuncture (ACP), recognized as a medical specialty by the Brazilian Federal Board of Medicine, is an option detailed in Traditional Chinese Medicine (TCM) and is based on need stimulation of specific points on the human body. Its use in symptoms such as tinnitus is similar to the model used in pain relief conditions, as both are reported as a disagreeable sensory and subjective emotional experience^2^.

Neuroscience studies related the effects of ACP to neuronal stimulus, activation of endogenous opioid mechanisms and neuropeptides which stimulate specific brain structures^3^.

Various ACP techniques have been described and the choice is based on the specificity and individuality of each proposed treatment. The aim of this study is to establish the efficacy of a specific ACP technique (scalp acupuncture) in the acute symptomatic relief of tinnitus.

## MATERIAL AND METHODS

A prospective randomized double-blind study of 76 patients seen at the Tinnitus Clinic of the Otorhinolaryngology and Head and Neck Surgery Department at UNIFESPEPM was conducted between April and June 2005.

**Figure 1:**
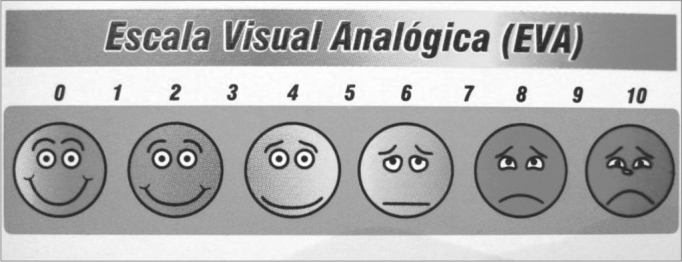
Visual analogue scale.

**Figure 2:**
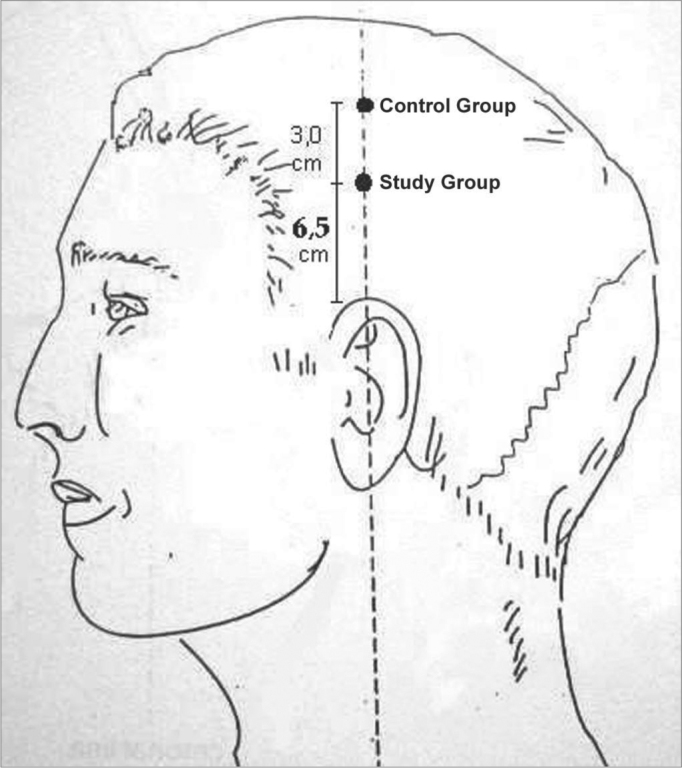
Points used.

**Figure 3:**
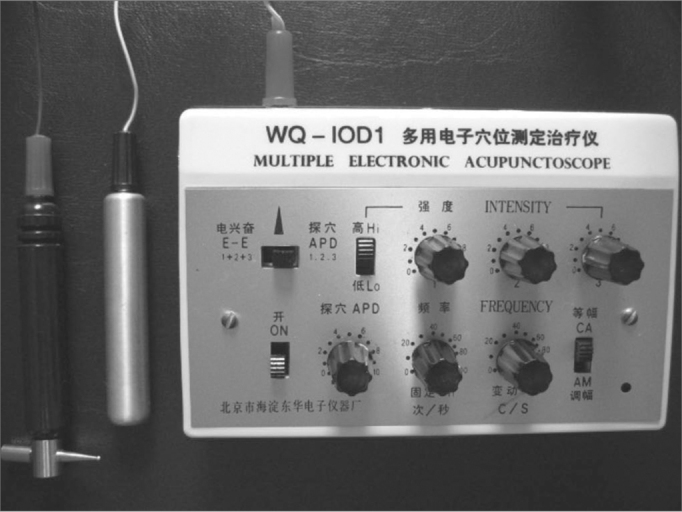
Point locating device.

All the patients complained of buzzing in the ears. The clinical history, physical examination and laboratory work-up were made to investigate the etiology of tinnitus. Patients then were sent to a first researcher to establish an initial numeric score from 0 to 10 for tinnitus based on the Visual Analogue Scale (VAS) (Figure 1). Patients with no tinnitus at this moment or that were unable to answer the questionnaire were excluded from the study. Patients were then sent to another office in which an acupuncture specialist and medical doctor, who had no access to the score, alternatively and sequentially allocated patients into one of two groups (control and study groups). Patients were placed in a silent room and needling was done on the side of the head where the patient complained of the loudest tinnitus. The ACP point used in the study group is located 6.5 cm above the apex of the ear pavilion in the temporoparietal region, which was identified with the help of an acupuncture point locating device. This point is the cochleal-vestibular area in scalp acupuncture, used to treat specifically a range of otological diseases in TCM.4 The point used in the control group was located 3 cm above the acupuncture point used in the study group, along the same vertical line, where the point locating device gave no characteristic signal and which does not correspond to any ACP points described by TCM (Figures 2 and 3).

**Figure 4:**
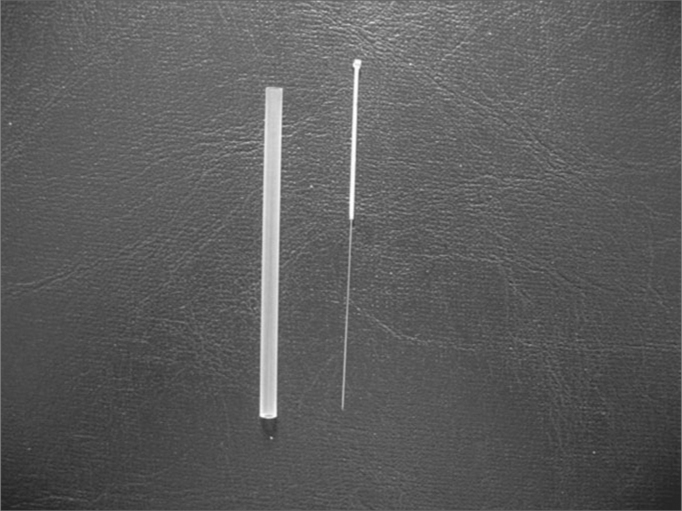
Needles used.

Stainless steel 0.3 x 40 mm disposable needles were inserted in the scalp to the periosteum at an angle of 45° (Figure 4). Patients remained in silence during one minute following manual rotating stimulus at 2 Hertz during 15 seconds. Patients were then sent to the first office for a second assessment by the first researcher, who did not know to which group the patient had been allocated. During this second interview patients were asked to redefine the tinnitus subjective score using the same scoring method as before.

After 5 days patients were contacted by phone to collect further data for the study, such as duration of relief and interference on the quality of sleep.

This protocol was evaluated and approved by the Ethics and Research Committee of UNIFESP-EPM and all patients included in the study read and signed an informed consent form.

Data were statistically analyzed using the analysis of variance (ANOVA) and independent t tests with a p<0.05 significance level. These tests are used when 3 or more information groups with numeric measurement levels are compared. Samples are independent and/or paired and the intention is to learn if on average the groups are different^4^.

## RESULTS

There were 76 patients in the study, 29 where men (38.2%) and 47 were women (61.8%). Average age was 56.9 ± 12.0 years. Each group (study and control) had 38 patients.

**Chart 1:**
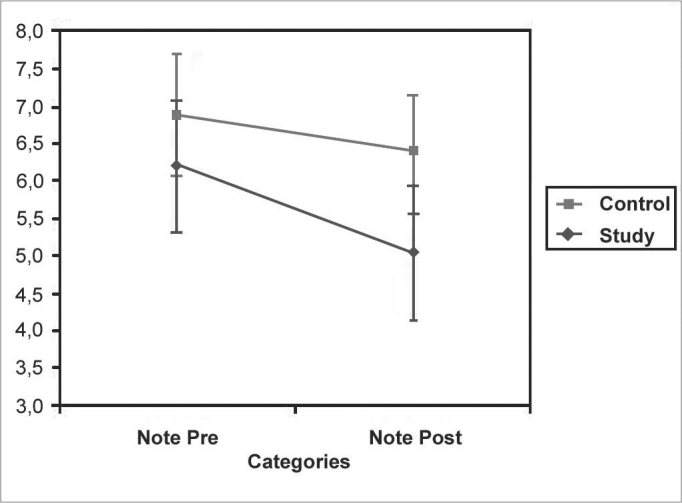
ANOVA test. Confidence interval for a mean: mean ± 1.96 × standard deviation / √ (n-1)

ANOVA demonstrated a significant difference (p<0.001) between pre and post needing moments and that patients in the study group showed greater relief of symptoms. This may also be seen in the significance of the interaction (p = 0.0127) (Chart 1).

**Chart 2:**
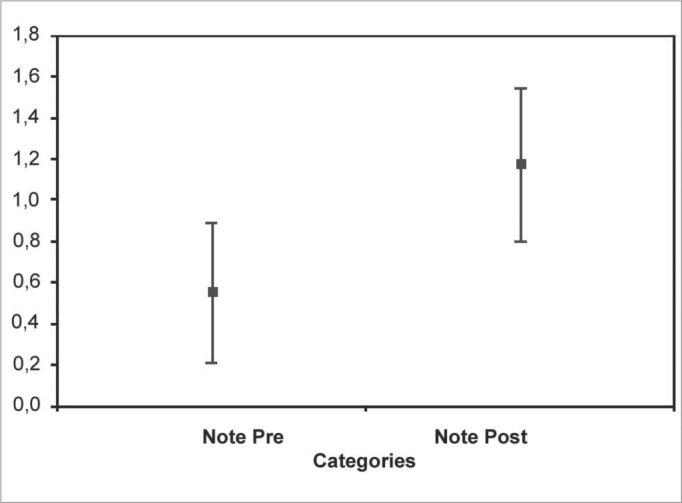
Independent t test. Confidence interval for a mean: mean ± 1.96 × standard deviation / √ (n-1)

The independent t test showed a significant difference (p=0.017) between pre and post needling scores in study and control groups (Chart 2).

Average duration of symptom relief (90.24 ±77.5 hours) varied from 106.9 hours in the study group to 72.3 hours in controls. Eight patients (10.5%) reported improved quality of sleep, 4 in the study group and 4 in controls.

Only 2 patients (2,6%) reported significant pain during needling. There were no reports of complications such as bleeding, infection or hematomas.

## DISCUSSION

Treatment of tinnitus with ACP is amply described in TCM^5^, ^6^. Scientific literature, however, lacks studies supporting its efficacy as a treatment option. ACP is a holistic form of treatment tailored to each individual. Thus protocols with adequate standardized methods that satisfy both TCM precepts and modern Western medicine are difficult to build. Therefore the choice was made to use a single craniopuncture point for symptom relief rather than cure.

Park et al. in 2000 conducted a systematic review and found 36 publications on this theme, but only six were randomized controlled studies. The author notes that there was a heterogeneous prescription of points and that results were controversial^7^.

Axelsson et al. in 1994 conducted a placebo-controlled study with 20 patients with tinnitus caused by noise, finding no significant difference in symptoms between groups. However, ACP points were not the same for all patients. They did note, however, improvement in the quality of sleep, muscle relaxation and blood circulation in the patients^8^.

Hansen et al. in 1982 conducted a double-blind study including 20 patients with unilateral tinnitus treated with 5 needles (on the head, feet and hands). Placebo was needles inserted into the subcutaneous tissue. The author used a diary of symptoms to compare both groups. There was no significant difference between groups^9^.

Marks et al. in 1984 conducted a similar study in 14 patients and found a negative evaluation of ACP in the treatment of tinnitus^10^.

On the other hand, Furugard et al. in 1991 conducted a study with 22 patients divided into treatment with ACP or physical therapy. They describe a 55% improvement in patients undergoing ACP needling. However, they noted that tinnitus returned to pre-treatment levels at one y ear follow-up^11^.

VAS was used by other authors to measure the level of buzzing reported by patients^7^, ^10^, ^11^. VAS is easy and quick to apply, making possible a comparison of results between groups.

This study shows that there is a symptom relief effect of tinnitus as a result of craniopuncture. Other non-specific factors, unrelated to cochleal-vestibular craniopuncture, may be related to this effect, such as induction by subjectivity on patients and the increased attention given by doctors to patients in the study.

The significant improvement levels following needling and the average duration of the effect justify the use of this technique. This study shows that this technique is safe and brings no side effects to patients. However, further studies are required to establish other possible effects of craniopuncture on the hearing system.

## CONCLUSION

There was a significant reduction in the score of pre and post needling moments in both groups (control and study groups). In the study group the reduction is greater compared to the controls.
